# Draft Genome Sequence of *Ornatilinea apprima* P3M-1, an Anaerobic Member of the *Chloroflexi* Class *Anaerolineae*

**DOI:** 10.1128/genomeA.01353-15

**Published:** 2015-11-19

**Authors:** James Hemp, Lewis M. Ward, Laura A. Pace, Woodward W. Fischer

**Affiliations:** a Division of Geological and Planetary Sciences, California Institute of Technology, Pasadena, California, USA; b Department of Medicine, University of California San Diego, La Jolla, California, USA

## Abstract

We report the draft genome sequence of *Ornatilinea apprima* P3M-1, a strictly anaerobic member of the *Chloroflexi* class *Anaerolineae*. This genome provides insight into the diversity of metabolism within the *Anaerolineae*, and the evolution of respiration within the *Chloroflexi*.

## GENOME ANNOUNCEMENT

*Ornatilinea apprima* P3M-1 was isolated in Siberia from a microbial mat in a wooden bathtub sourced with water from a 2,775-m well (1). Closely related strains have been reported from fresh water lakes and rice paddy soils. *O. apprima* was physiologically characterized as a filamentous, nonmotile, obligately anaerobic organotroph. It can ferment a wide range of polypeptides and carbohydrates, including microcrystalline cellulose. It grows optimally at 42 to 45°C and pH 7.5 to 8.0 (1).

The genome of *Ornatilinea apprima* P3M-1 (DSM 23815) was sequenced as part of a project to expand the phylogenetic breadth of *Chloroflexi* genomes. Genome sequencing was performed at Seqmatic using the Illumina MiSeq sequencing platform. SPAdes version 3.1.1 (2) was used to assemble the genome. The genome was screened for contaminants based on sequence coverage, GC composition, and BLAST hits of conserved single-copy genes. Genome annotation was performed using the NCBI Prokaryotic Genome Annotation Pipeline. The draft genome is 4.41 Mb in size, assembled into 45 contigs. It encodes 3,846 genes, 3,347 coding sequences, 2 16S RNAs, 50 tRNAs, and 3 CRISPR arrays. It is estimated to be ~96% complete, based on conserved single-copy genes (107/111).

The majority of cultured *Chloroflexi* belong to the class *Chloroflexia*, which is composed of anoxygenic phototrophs and facultative aerobes (3). Even though >70% of all *Chloroflexi* sequences present in 16S datasets belong to the *Anaerolineae* class (4), they are less well characterized. To date, all described members are anaerobic fermentative organisms (5). Consistent with its description as an obligate anaerobe, *O. apprima* contains no genes for O_2_ respiration. Even though it was classified as nonmotile, the genome does encode genes for flagella and chemotaxis, two traits not previously observed in *Anaerolineae*, suggesting that it is capable of motility*.* In addition, no genes for LPS biosynthesis or outer-membrane proteins were found, which is consistent with the hypothesis that *Chloroflexi* have only one membrane (6).

### Nucleotide sequence accession number.

This whole-genome shotgun project has been deposited in DDBJ/EMBL/GenBank under the accession number LGCL00000000.
